# The Potential of Selenium-Based Therapies for Ocular Oxidative Stress

**DOI:** 10.3390/pharmaceutics16050631

**Published:** 2024-05-08

**Authors:** Lulwah Al-Bassam, Gemma C. Shearman, Steve Brocchini, Raid G. Alany, Gareth R. Williams

**Affiliations:** 1 UCL School of Pharmacy, University College London, 29-39 Brunswick Square, London WC1N 1AX, UK; lulwah.albassam.20@ucl.ac.uk (L.A.-B.); s.brocchini@ucl.ac.uk (S.B.); 2 School of Life Sciences, Pharmacy and Chemistry, Kingston University London, Penrhyn Rd, Kingston upon Thames KT1 2EE, UK; g.shearman@kingston.ac.uk (G.C.S.); r.alany@kingston.ac.uk (R.G.A.); 3 School of Pharmacy, The University of Auckland, Auckland 1142, New Zealand

**Keywords:** antioxidant, ocular conditions, oxidative stress, selenium, selenoprotein, glutathione peroxidase

## Abstract

Oxidative stress plays a critical role in the development of chronic ocular conditions including cataracts, age-related macular degeneration, and diabetic retinopathy. There is a need to explore the potential of topical antioxidants to slow the progression of those conditions by mediating oxidative stress and maintaining ocular health. Selenium has attracted considerable attention because it is a component of selenoproteins and antioxidant enzymes. The application of selenium to a patient can increase selenoprotein expression, counteracting the effect of reactive oxygen species by increasing the presence of antioxidant enzymes, and thus slowing the progression of chronic ocular disorders. Oxidative stress effects at the biomolecular level for prevalent ocular conditions are described in this review along with some of the known defensive mechanisms, with a focus on selenoproteins. The importance of selenium in the eye is described, along with a discussion of selenium studies and uses. Selenium’s antioxidant and anti-inflammatory qualities may prevent or delay eye diseases. Recent breakthroughs in drug delivery methods and nanotechnology for selenium-based ocular medication delivery are enumerated. Different types of selenium may be employed in formulations aimed at managing ocular oxidative stress conditions.

## 1. Introduction

Recently, the World Health Organisation (WHO) estimated that there are almost 2.2 billion individuals globally who have visual impairment, and it is thought that half of these instances could be avoided [1]. The leading cause of vision disability amongst the elderly is age-related impairment caused mainly by cataracts, age-related macular degeneration (AMD), glaucoma, and diabetic retinopathy (DR) [2]. Oxidative stress is a critical factor in the progression of these conditions [3]. With increases in age expectancy, more individuals will suffer from vision loss, affecting the quality of their lives and overall wellbeing [2]. Therefore, the development of treatments to slow the progression of blinding ocular conditions remains an important unmet medical need.

This review provides an insight into age-related ocular conditions where oxidative stress is a contributing factor. Possible treatment options and impacts of selenium (Se) as an antioxidant to manage oxidative stress will be presented. The potential efficacy of Se to cause the retrogression of several ocular conditions such as cataracts and DR will be considered.

A systematic review of the literature was performed to gain insight into the protective and antioxidant effects of orally and topically administered selenium-based formulations. We searched Scopus, PubMed, Web of Science, and Google Scholar using the following terms: “selenium”, “ocular health”, “eye diseases”, “cell studies”, “in vitro”, “animal models”, “rats”, “mice”, and “clinical trials” to ensure comprehensive coverage. Articles not in English were excluded from consideration. Types of studies considered include randomised controlled trials (RCTs), cohort studies, case–control studies, in vitro cell culture studies, and animal studies employing rats, mice, and other relevant models. Studies exploring the relationship between selenium consumption and ocular diseases were selected.

## 2. Basic Anatomy of the Eye

**Figure 1:**
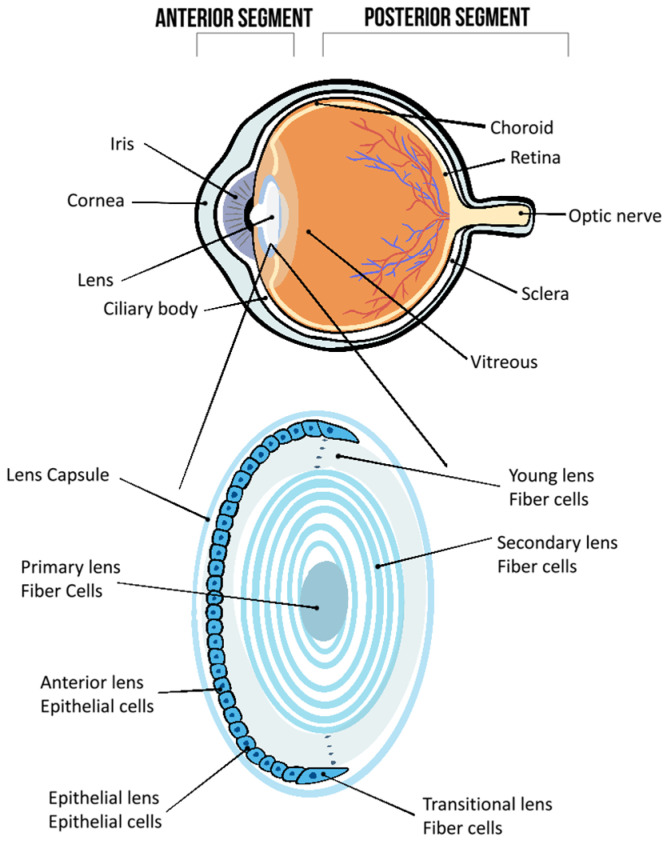
Top: the anterior and posterior ocular segments of the eye, adapted from [7]. Bottom: a cross-section of the human eye lens showing layers of lens fibre cells, the epithelial cell layer (metabolically active site, containing all cell organelles), and the lens nucleus. Mature fibre cells in the cortex elongate and undergo de-nucleation moving towards the nucleus, adapted from [8].

The eye represents a sophisticated sensory apparatus consisting of anterior and posterior chambers. The anterior part covers the tear film, cornea, sclera, iris, ciliary body, and lens [4] (Figure 1). The tear film functions to lubricate the eye’s surface, provides oxygen and nutrients to the cornea, and covers the corneal surface, creating an ideal medium for light refraction in the eye [5]. The cornea is the transparent, most anterior, part of the eye. The cornea presents major physical and biochemical barriers that act as a significant avascular barrier for drug absorption into the anterior chamber of the eye [4]. The sclera supports the retina and safeguards eye components. Its opaqueness also blocks light from angles that might impair the quality of the retinal picture [6]. The iris modulates the amount of light that reaches the retina by controlling the diameter of the pupil. The ciliary body, which comprises ciliary muscles, epithelium, and stroma, controls the lens’s power and shape. It is also the site of aqueous production, which is the main mass transfer mechanism of the eye [4]. Aqueous outflow through the anterior chamber into the trabecular meshwork (TM) (and Schlemm’s canal) is important to maintain the health and function of the avascular elements of the eye which must remain transparent (e.g., cornea, lens).

The lens is composed of a capsule, epithelium, and cortex surrounding the fibrous nucleus [7] (Figure 1). The lens capsule is an elastic tissue, adapting to shape changes and protecting the lens from infections. The outer cell layer of the lens comprises a single layer of epithelial cells, which are the only metabolically active element inside the lens, containing the majority of transporters and metabolic enzymes in the lens [7,8]. Mitosis generates new cells in the epithelial layer, which eventually differentiate into fibre cells [9]. These fibre cells express crystallin proteins, which determine the refractive index and transparency of the lens [7]. The developed fibre cells travel toward the centre of the lens, where they de-nucleate and lose their organelles and cellular features [9]. Eventually, these cells stack into numerous layers surrounding the lens’ central nucleus, producing the cortex [9,10]. The lack of organelle-like sub-structure during fibre cell maturation leads to a very low capacity for protein turnover and no capacity to express new proteins [7]. Since there is minimal protein turnover in the lens fibre cells, crystallin proteins are required to endure throughout an individual’s lifetime. Thus, throughout life, the human lens grows at a slow rate without tissue loss, aggregating old fibres in the lens [7]. Fibre crystallins are long-term stable proteins and considered to be molecular chaperones that protect the lens against physiological stress [7,10]. The crystallins are however subjected to oxidative stress that result in the modification and aggregation of those proteins [7].

The posterior cavity of the eye consists of the retina, macula, choroid, vitreous humour, and the optic nerve [11]. The retina is a highly complex neurosensory tissue composed of neurons, nerve fibres, and glial cells. There are six types of neurons, which transduce light to an electrical signal to the brain via the optic nerve. The macula is a pigmented region in the retina that is rich in photoreceptors and responsible for precise central vision [11,12]. The choroid is a vascular layer responsible for delivering oxygen and nutrients to the retinal layers. The vitreous humour is a clear gel which helps to maintain the globe of the eye and is composed of water (~98%) and collagen II, hyaluronic acid, and glycosaminoglycans [11].

## 3. Oxidative Stress and Age-Related Effects on the Anterior Segment of the Eye

**Figure 2:**
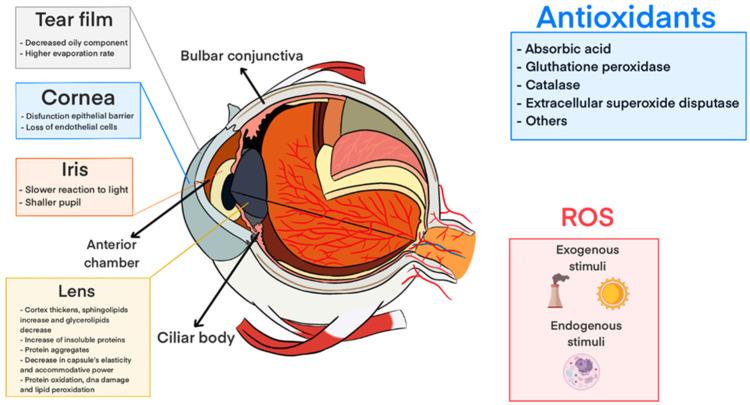
Effects of oxidative stress on different parts of the anterior segment of the eye, adapted from [15].

With aging, antioxidants such as glutathione and ascorbic acid become depleted and the eye becomes more susceptible to a variety of conditions as a result of oxidative stress [13]. The continuous exposure of the lens to ultraviolet (UV) radiation along with other exogenous factors (e.g., environmental pollution and smoking) leads to the generation of reactive oxygen species (ROS), which non-specifically undergo reactions with proteins and lipids of the lens crystallins, leading to lipid peroxidation (LPO) and uncontrolled damage of the lens cell membrane [10]. With time, the lens increases in weight and thickness, creating a barrier to antioxidant transport between the lens epithelium and the nucleus [8]. Furthermore, many post-translational modifications (PTMs) of crystallin proteins, including cross-linking, denaturation, deamidation, glycation, and oxidation, occur with aging. All these processes result in crystallin aggregation, loss of enzyme activity, and disruption of ion transport throughout the lens (Figure 2) [10,14]. Since the crystallin proteins of the lens last a lifetime, any age-related damage has a cumulative effect [8]. With time, these changes result in increased light-scattering and a loss of lens transparency and elasticity, which leads to individuals experiencing blurry vision with the onset of cataract formation.

ROS can also damage the tear lipid layer, initiating the release of inflammatory mediators that cause cell damage and disturb tear film stability within the lipid layer [16]. Dry eye is the loss of tear film homeostasis in the ocular surface, leading to tear hyperosmolarity (evaporation of the aqueous layer of the tear film from the exposed ocular surface during low aqueous tear flow) and causing a series of signal-inducing oxidative stress markers [17,18,19]. LPO secondary products 4-hydroxynonenal (4-HNE) and malondialdehyde (MDA) were found to be elevated in a primary human corneal epithelial cell line from human donors (aged 16 to 67 years), and antioxidant enzymes were reduced, indicating that tear hyperosmolarity induces oxidative stress [20]. A case–control study showed that patients with dry eye expressed higher concentrations of MDA and 4-HNE in the conjunctiva than the controls, indicating that dry eye condition can be caused by ROS [21].

Oxidative stress could also be a contributing factor in Fuchs’ endothelial cell dystrophy pathogenesis. The corneal epithelium, populated with mitochondria, is exposed to light and high oxygen levels, creating a rich ROS environment [22,23]. Moreover, oxidative stress contributes to the breakdown of basic intracellular molecules and alters the synthesis of collagen and elastin. It therefore results in pterygium, which is an irregular growth of epithelial tissue on the conjunctiva proliferating into the cornea [24]. A study found that 4-hydroxy-2-nonenal (4-HHE) and (4-HNE) were concentrated in pterygial specimens, and elevated levels of MDA were observed, signifying oxidative stress [24]. Two studies detected 8-hydroxy-2′-deoxyguanosine (8-OHdG) in pterygium samples, indicating that oxidative DNA damage by ROS had occurred [25,26]. Furthermore, Kormanovski et al. indicated that oxidative stress plays a role in pterygium recurrence [27].

## 4. Oxidative Stress and Age-Related Effects on the Posterior Segment of the Eye

Oxidative stress can cause functional and structural damage in the retinal pigment epithelium, endothelial cells, and retinal ganglion cells (RGCs). Oxidative stress appears to play a critical part in initiating blinding retinal conditions such as AMD, glaucoma, and DR [28].

AMD is a progressive condition affecting the macula, marked by central vision decline attributed to abnormalities in both photoreceptors and retinal pigment epithelium (RPE) [11,17,18,29]. With age, the buildup of drusen (accumulation of yellowish extracellular lipid and protein deposits of cellular debris origin) and high levels of advanced glycation end products (AGEs) results in an ideal environment for ROS and LPO generation, since the retina consumes high levels of oxygen supplemented via choroidal vasculature and retinal circulation. Additionally, the retina is sensitive to light, leading to the formation of LPO from outer segment photoreceptor membranes that are rich in polyunsaturated fatty acids (PUFAs) [11,17,18,29]. AMD patients showed significantly higher levels of MDA and 8-OHdG and damaged DNA biomarkers, and analysis of their blood serum indicates elevated oxidative stress compared to healthy controls [30]. Further studies showed other elevated oxidative stress markers and mitochondrial malfunction in the retina of patients with AMD [18,31,32].

Oxidative stress results in retinal vascular and neuronal damage such as hyperpermeability, apoptosis, angiogenesis, and retinal tissue damage, which are correlated with DR [11,33,34,35,36,37,38]. A case–control study showed that patients with DR had a significant increase in thiobarbituric-acid-reacting substances and LPO compared to the controls, suggesting an elevated level of LPO has a strong association with DR [38]. Another study demonstrated that the serum MDA and advanced oxidised protein products of DR patients were increased compared to patients with non-diabetic eye disorders [39].

Glaucoma is a chronic visual neuropathy characterised by retinal ganglion cell degeneration and, hence, the deformation of the optic nerve head [40,41]. Patients with glaucoma showed elevated levels of MDA and a reduced level of total antioxidant capacity in the blood and aqueous humour samples compared to non-glaucomatous patients [42]. Recently, a case–control study established that glaucoma patients experienced a decrease in total antioxidant status and superoxide dismutase (SOD) levels, in addition to an increase in MDA levels compared to the controls [43]. Experimental data also demonstrated that, under oxidative stress, local T-cells might be activated within the optic nerve head due to the increased antigenicity of supportive glial cells. This stimulation of an adaptive immune response has the potential to cause neurodegeneration in the optic nerve [44].

ROS can promote apoptosis and inflammatory pathways in the TM. High ROS levels here can provoke the structural and functional damage of mitochondrial–DNA components, proteins, and membrane lipids. Furthermore, elevated ROS levels affect the TM nuclear factor–κB (NF-κB) pathway, causing oxidative stress. In addition, ROS causes the apoptosis of retinal ganglion cells, optic nerve glial malfunction, and disrupts ophthalmic physiology [25,26,45].

## 5. Defensive Mechanisms to Minimise Ocular Oxidative Stress

The antioxidant defence system is composed of enzymatic and non-enzymatic molecules. The key non-enzymatic antioxidant defence mechanism in the eye is mainly performed by ascorbic acid (AA) and glutathione (GSH) [8]. AA has a scavenging effect as it is a substantial electron donor that can undergo reaction with ROS [46]. AA is oxidised by free radicals to ascorbate free radicals and dehydroascorbate (DHA), which can be reduced back to AA with the aid of GSH or nicotinamide adenine dinucleotide phosphate (NADPH) [46]. Humans, however, lack the enzyme L-gluconolactone oxidase to synthesise AA. Thus, a dietary supply of AA is critical [46].

Glutathione is mainly synthesised from cysteine, glutamate, and glycine [46]. Distinct from AA, GSH in the lens epithelium and outer cortex is synthesised via a de novo biosynthetic pathway [3,10]. The antioxidant capacity of glutathione is due to the thiol group, which shows reactivity towards electrophiles and oxidants. It acts as a reducing equivalent, thus protecting cellular protein thiols from oxidation [47].

α-Tocopherol (vitamin E) is essential for the preservation of cellular membrane integrity, being the principal lipid-soluble defence antioxidant in membranes. It is a fat-soluble antioxidant that works exclusively by quenching peroxyl radicals to disrupt the chain reaction of LPO [3]. α-Tocopherol donates a proton to peroxyl radicals, forming a tocopherol radical that reacts with another peroxyl radical to give non-reactive products [48].

**Figure 3:**
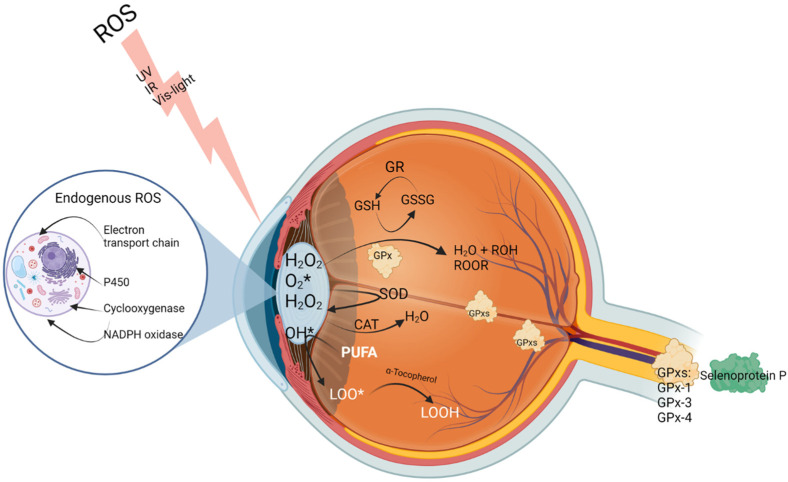
Oxidative stress and scavenging processes in the eye. ROS sources can be endogenous or exogenous, creating superoxide anions (which can be reduced by SOD). Hydrogen peroxide can cross cell membranes easily and cause further oxidation. Hydroxyl radicals further react with cell lipids and nucleotides, leading to damaged membranes, proteins, and lipids, as well as DNA. GPx catalyses reactions of peroxides, using GSH [50]. * denotes radicals. Image created with Biorender.

In the anterior segment of the eye, the glutathione-related enzymes catalase (CAT) and SOD are present to regulate ROS production and perform scavenging functions (Figure 3) [46,49]. The GSH antioxidant enzyme reduces hydrogen peroxide (H_2_O_2_), with glutathione peroxidase (GPx) catalysing the oxidation of GSH to glutathione disulfide (GSSG). The reduction of H_2_O_2_ to hydroxide and water breaks the peroxide-dependent free radical reaction-dependent oxidative stress process (Figure 3) [3,47]. Together with GPx, glutathione reductase accelerates the conversion of GSSG to its reduced form in the presence of NADPH to maintain the level of GSH [46].

GPx is a critical intracellular enzyme that protects lipids from peroxidation [51]. This cytosolic enzyme helps to retain the structure and function of the cell [49]. GPx is composed of four identical subunits containing a selenium atom (from diet) integrated in the form of selenocysteine (Sec) and a GSH binding site. There are eight isoforms in the human GPx family. The ocular surface mostly expresses GPx1 and GPx4 and GPx3 in the sclera [49]. The lens expresses the GPx1, GPx3, and GPx4 isoforms.

**Figure 4:**
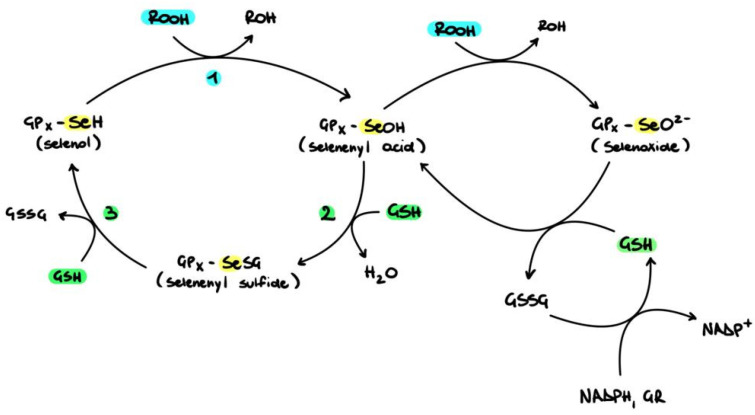
Redox cycle of glutathione peroxidase (GPx). (1) The SeH in the GPx can be oxidised by H_2_O_2_ or other oxidants to selenic acid (GPx-Se-OH). This can then be transformed back to (GPx-SeH) via two steps. First, (2) glutathione reduces GPx-Se-OH to GPx-SeSG, and then (3) this is reduced back to GPx-SeH. GPx-Se-OH can also be overoxidised to seleninic acid (GPx-SeO_2_H) due to levels of oxidative stress or low glutathione concentrations. Adapted from [51].

Within the cellular membrane, GPx4 reduces fatty acid hydroperoxides and phospholipid hydroperoxides working in combination with α-tocopherol to prevent LPO (Figure 4) [52]. The primary intracellular isoform is GPx1, the absence of which has been associated with membrane damage in the lenticular nucleus [49]. Gene-knockout mice with GPx1 deficiency exhibited increased nuclear light scattering in the lens, membrane damage, and cataract development, demonstrating the significant role of GPX-1 in the antioxidant defence systems of the lens nucleus [53]. The significance of GPx activity in cataract development (particularly nuclear cataract) is well established [54,55,56]. Antioxidant levels become significantly depleted with age, resulting in oxidative stress and damage to different ocular segments [13]. Therefore, supplementary antioxidants are used in an effort to counteract the loss of some ocular antioxidants and slow down the progression of ocular conditions. Dietary Se is fundamental to ensure sufficient GPx expression.

## 6. Selenium and Selenoproteins

Selenium (Se) is an essential trace element existing in different oxidation states: elemental state (Se^0^) (0), selenides (Se^2−^) (−2), selenites (SeO_3_^2−^) (IV), and selenates (SeO_4_^2−^) (VI) [57]. The chemical form of Se (organic/inorganic/elemental) influences its bioavailability and toxicity [58]. Selenium levels vary in human serum with age, sex, and geographical region [59]. This variation likely arises from differences in the amount of selenium (organic and inorganic) in food, water, and soil [60]. Seafood, muscle meats, cereals, grains, and dairy products are some of the foods that are high in total selenium [57]. Selenium serum levels among Europeans are on average 85.19 ± 14.58 µg/L for people above 19 years old [59]. According to the UK NHS, individuals aged 19 and above need a daily intake of 75 µg of selenium for males and 60 µg for women [61]. In the UK over the last 20 years, Se intake has dropped, and recent surveys show that the average Se intake is now only 30–40 µg per day [62]. Severe Se deficiency has adverse biophysiological effects, such as infertility, weakness, and fatigue [63].

**Figure 5:**
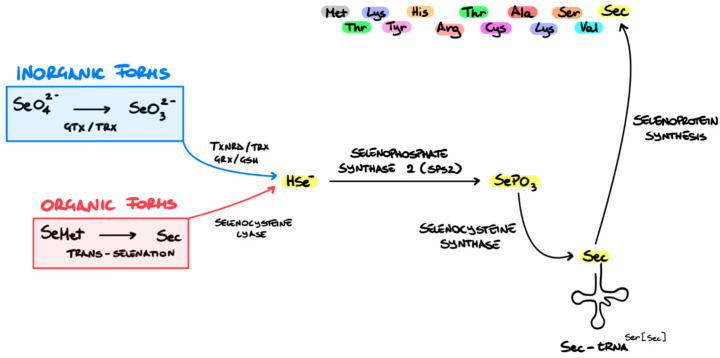
Selenoprotein biosynthesis. Nutritional Se, regardless of its form, must be transformed to the active metabolite selenide (Se^2−^), which serves as the selenium pool for the integration of selenium into selenoproteins. Inorganic Se forms are converted to the active metabolite via the GSH–GR and thioredoxin pathways, while organic forms are metabolised by selenocysteine lyase. Then, tselenophosphate (SePO_3_^3−^) becomes incorporated into proteins via a distinctive pathway where a seryl (Ser) residue is exchanged with selenocysteine (Sec) before integration into proteins. Adapted from [67].

Selenium is fundamentally important to human wellbeing in its organic forms, which include selenomethionine (Se-Met), selenocysteine (Sec), and methyl selenocysteine [64]. Selenium metabolism, including absorption, transport, distribution, excretion, retention, and conversion to the active metabolite, is highly reliant on the chemical form and amount of selenium consumed [65]. Dietary Se (organic or inorganic) is metabolised by different routes (depending on its form) to yield hydrogen selenide (H_2_Se) or selenide (Se^2−^). H_2_Se is the active metabolite and serves as the selenium pool for Sec biosynthesis, which is the main route for selenoprotein synthesis (Figure 5) [66]. Therefore, inadequate selenium consumption results in the depression of selenoprotein synthesis [65].

There are 25 human selenoproteins that have been identified, which include the GPx family, selenoprotein P, and thioredoxin reductases [68]. In selenoproteins, Se is substituted for sulfur in cysteine [69]. Se is a more reactive nucleophile than sulfur, providing 1000-fold enhanced enzymatic activity than cysteine homologs [63,68,69,70]. Under oxidative stress, hydrogen peroxides are likely to react with selenol-containing amino acids, such as those in GPx, before other potential targets (thiol-containing proteins) [69]. The oxidised form of a selenoprotein is rapidly reduced and recycled, and hence rapidly removes H_2_O_2_. In contrast, thiol-containing proteins are reduced at a slower rate, primarily via NADPH-driven enzymatic reactions.

The administration of supplemental selenium has the potential to optimise the expression and functionality of selenoproteins and enzymes, enabling them to achieve their maximum efficacy [71,72,73]. Nevertheless, there is a widespread belief that the administration of selenium compounds in doses exceeding the recommended levels can potentially result in toxic effects [74,75]. However, several studies have provided evidence for the existence of benefits associated with elevated levels of selenium, while also reporting limited adverse effects [76,77].

It is noteworthy to mention that when selenium is supplemented at higher concentrations, the expression of selenoenzymes does not surpass levels that align with the acceptable daily intake for humans [71,72]. The supplementation of Se can be saturated; hence, further investigations are warranted to examine the potential benefits of administering high doses of selenium in the context of e.g. cancer therapy. The metabolic by-products of substances containing selenium have the potential to facilitate the generation of hydrogen peroxide by means of superoxide synthesis. Moreover, they have the potential to participate in the oxidation of cysteine, resulting in the occurrence of DNA damage [78,79].

Elemental selenium (0) is insoluble and is typically considered to be biologically inert [80]. However, nanotechnology shows potential in the domains of medication and nutrition due to the advantageous properties displayed by materials at the nanometre scale, which differ from both individual atoms and bulk materials [81]. Selenium nanoparticles (SeNPs) (<100 nm) have been the focus of recent research due to their reduced toxicity compared to other forms of selenium (organic/inorganic) [58,64,82]. The advantage of SeNPs lies in their size and the presence of selenium in the elemental form (Se^0^), which shows low toxicity compared to other oxidation states (Se^IV^, Se^VI^) [64]. The range of acute toxicity of SeNPs is seven times lower than for sodium selenite and four times lower compared to selenomethionine (SeMet) in [83,84]. The antioxidant and pro-oxidant effects of elemental Se, depending on its concentration, require that selenium be considered with some caution [64]. Selenium’s toxicity is attributed to its pro-oxidant capacity, which enables it to catalyse the oxidation of thiols and therefore produce ROS [76,78,79]. Comparing the oxidation potential of the different forms of selenium depends on the reaction conditions. Inorganic selenium (Se^IV^, Se^VI^) requires two steps of reduction (oxidizing GSH) to be transformed to the active metabolite, while the organic form is metabolised by selenocysteine lyase via a process that does not include the oxidation step [67]. Elemental selenium in the nano size, on the other hand, has an unclear path of metabolism due to the much reduced particle size that does not resemble the bulk material and follows the mechanism of nanoparticles uptake and metabolism [85].

Several studies compared different forms of Se to explore biocompatibility and antioxidant effects [80,84,86]. In this regard, Wang et al. [84] compared the effects of SeNPs with SeMet on mice. Both led to a significant increase in GPx activities in the plasma, liver, and kidney. However, SeNPs were found to have greater efficacy in increasing GPx levels in the liver than SeMet. Zhang et al. [80] obtained similar results when they compared the antioxidant effects of elemental SeNPs with Se-methyl-selenocysteine (SeMSC). These outcomes were consistent with work by Boostani and co-workers [86], who studied farmed chickens that were exposed to oxidative stress by the peritoneal administration of tert-butyl hydroperoxide, and then monitored their GSH levels. GSH was elevated in the SeNPs group as well as the organic selenium group compared to the control group (corn–soybean-fed chickens). Moreover, the GPx concentration was reported to be highest in the SeNPs group (80 units/mg protein). Due to differences in the absorption and metabolism of the various Se forms, they demonstrate different bioavailability and toxicity [78,79,80].

**Figure 6:**
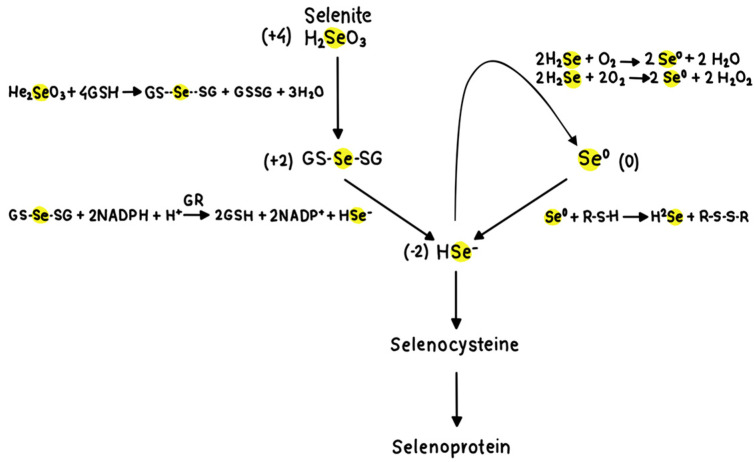
Pathways for selenide formation and selenocysteine (SeCys) production. Elemental Se can be reduced to selenide (active metabolite) via reaction with sulfur compounds (e.g., cysteine) R-SH. Se^0^ + 2 R-S-H → H_2_Se + R-S-S-R. Adapted from [87].

The efficiency with which SeNPs can be metabolised from the diet and then integrated in reduced form into selenoproteins is unknown. However, the studies mentioned above show an increase in GPx enzyme activity in response to dietary SeNPs, confirming elemental Se metabolism and its utilisation to synthesize selenoproteins. Recently, a study compared the bioavailability of SeNPs with other Se forms (sodium selenite and SeMet) in mice [87]. Elemental SeNPs increased the GPx enzyme activity levels in Se-fed mice similarly to those caused by organic Se (SeMet) and to a greater extent than sodium selenite. This could be due to the fact that the conversion of elemental Se (0) to hydrogen selenide (−2) only requires one reduction step, but the reduction of selenite (+4) to hydrogen selenide requires numerous processes (Figure 6). This study also pointed out that since both elemental Se and inorganic Se were incorporated into selenoproteins, there is likely to be a common metabolic pathway. Elemental Se can readily react with sulfur compounds, yielding selenium in the fully reduced form, hydrogen selenide, which is the active metabolite for selenoprotein synthesis (Se^0^ + 2 R-S-H →H_2_Se + R-S-S-R) [87].

Other selenoproteins include selenoprotein P, a glycoprotein which mainly transports selenium to remote tissues and is responsible for approximately 50% of the Se concentration in the plasma (comprising all selenoproteins, selenocysteine, and selenomethionine) [52,66,88]. There is also thioredoxin reductase, which aids in regenerating AA from dehydroascorbic acid [65]. The suitable range of selenium in plasma may be between 60 and 150 ng/mL [89], although there is no agreement for a recognised standard for the plasma selenium concentration in healthy individuals.

## 7. Selenium Effects on Ocular Conditions

There are a limited number of published studies describing topical selenium effects on ocular conditions. Higuchi [88] and co-workers formulated selenoprotein P (SeP) eye drops, isolated through human serum fractionation, to test the oxidative damage on a Se-deprived human corneal epithelial cell line (CEPI) and to treat dry eye in a rat model. Selenoprotein P is produced by the lacrimal gland and secreted in tears in order to provide selenium to the corneal epithelium. Initially, the cultured cells showed high oxidative stress marker production (8-OHdG) and reduced GPx activity. However, upon the administration of SeP drops to the medium, the researchers observed the full recovery of GPx activity, and thus 8-OHdG, HEL, and LPO production were significantly suppressed by GPx. Then, SeP eye drops were administered in a dry eye rat model, and the cornea was assessed using the fluorescein score, which detects foreign bodies or damage [88,90]. A higher score indicates a greater amount of damage. The fluorescein score in this study was low compared to what is observed in healthy rat corneas. This study also compared the SeP concentration in human tears collected from healthy volunteers to dry eye patients. The average SeP content in the tears of the dry eye patients was 56 ± 30 ng/mL, while it was 93 ± 57 ng/mL in healthy volunteers. This is suggestive that a decrease in the selenium level may be correlated to oxidative stress disorders, though causation cannot be established from the data presented [88]. A recent study developed a topical formulation combining copper selenium nanoparticles with aldehyde-factionalised Pluronic F127 to treat dry eye [91]. This study demonstrated that a concentration of 0.1 µg/mL of the nanoparticles effectively eliminated intracellular ROS and increased the activity of GPx and SOD in human corneal epithelial cells.

Another study evaluated the ability of different Se compounds to protect the corneal epithelium against oxidative stress [92]. The authors prepared a topical eye drop containing Se and lactoferrin. The latter is a biological iron-chelating glycoprotein that has a protective effect on the corneal epithelium. The effect of different selenium compounds (sodium selenite, SeMet, selenocysteine, selenium containing peptides, and Se–lactoferrin) on the Se-deprived CEPI cell line’s viability was assessed, revealing that sodium selenite (an inorganic form) had the highest toxicity [92]. Furthermore, the therapeutic effects of selenium compounds were evaluated using a dry eye rat model. Notably, not all Se-containing compounds showed efficacy, but the Se–lactoferrin eye drops (18 μM) led to the significant suppression of 8-OHdG production, suppressed corneal irritation, and resulted in the significant amelioration of corneal damage caused by dry eye. Additionally, in dry eye models, there was an increased level of oxidative stress markers in the rat corneas which decreased upon Se–lactoferrin eye drop administration, consistent with a reduction in oxidative stress levels. Se-containing topical formulations could, hence, be candidates to manage oxidative-stress-related conditions like dry eye [92].

A comparative study between Se–lactoferrin and diquafosol tetrasodium (a topical eye drop used to manage dry eye) was conducted to evaluate their abilities to treat dry eye in rat and rabbit models. Higuchi et al. [93] showed that administering 0.1% Se–lactoferrin (containing 18 μM of Se) decreased the ocular fluorescein score to half compared to the diquafosol tetrasodium score in a rat model [93]. In addition, upon the administration of 0.1, 0.5, or 1.0% *w*/*v* Se–lactoferrin (containing 1.8, 18, or 180 µM Se, respectively) in albino rabbits’ eyes, no ocular irritation was observed [93]. The Se topical formulations studied to date are summarised in Table 1.

Some studies have described the effects of orally administered selenium supplements on the lens and age-related cataract formation (Table 2). A low serum level (less than 70 μg/L) of Se was associated with cataract formation [70]. The recommended serum Se levels in humans range between 78.9 µg/L and 94.7 µg/L, which is ideal for GPx and other selenoprotein functions [70]

In another study, dietary Se counteracted mercury (Hg) toxic effects on the lens in individuals that consume a Hg-rich diet [94]. Also, oral Se supplements (sodium selenite less than 0.04 mg/kg/day) reduced cataract progression in rats by increasing GPx activities in the blood and the lens [95]. Furthermore, sodium selenite showed a prophylactic effect against oxidative stress, restored GSH, and decreased MDA levels in rats [95]. However, the study showed signs of oxidative stress at a dosage of 0.04 mg/kg/day, highlighting the need to further explore the therapeutic range.

Some cohort studies have focused on the effects of orally administered Se (either in organic or inorganic forms) on cataract formation and progression, generally showing only slight benefits in humans (Table 2). For instance, one study showed that orally administered Se (35 μg/day, inorganic Se within a Centrum vitamin tablet) could decrease nuclear opacity [96], while other studies showed no significant effect of L-selenomethionine supplementation or dietary Se on cataract formation [97,98]. The variation in these results may be due to factors including differing Se doses, gender differences, different cataract opacification classification systems, and other supplements used with Se. Generally, intermediate doses of the dietary allowance of Se (60–70 µg/day) are more protective for the lens than higher doses [99]. A study was carried out on a human lens epithelial (HLE) cell line that evaluated the effect of selenium nanoparticles on the lens after UVB damage. Nano-selenium loaded with different concentrations of lycium barbarum polysaccharide was tested to determine its effect on cell survival; LBP-SeNPs can protect the HLE cell line from UVB-induced damage, and the cell proliferation rate is further increased with a decreasing nano-selenium concentration. Further studies are underway to establish the most effective concentration that improves cell survival [100].

Excessive Se intake (more than the recommended daily dose) results in promoting ROS production through generating peroxides (Figure 6) [101]. This excess amount of Se, regardless of its form, results in more of the hydrogen selenide metabolite, which can undergo reaction with oxygen, forming more H_2_O_2_ (Figure 6) [87]. For instance, the excessive intake of selenium will in turn lead to the formation of cataracts, known as the selenium cataract [95].

**Table 2:** Studies on oral formulations showing the effect of selenium on cataract development. Most studies showed correlation between low Se concentrations and cataract formation, and that Se can serve as a protective factor.

Study	Indication/Goal	Study Form	Results
Christen W (2015)	To test Se effects on inducing cataracts	Serum blood test L-selenomethionine (oral supplement)	No correlation was established [97].
Lemire M (2010)	Evaluate Se influence on age-relate cataracts (ARC)	Serum blood test (dietary)	ARC was correlated with low Se levels [94].
Li T (2008)	To test Se effects on inducing cataracts	Cross-sectional study of 1522 persons aged ≥ 50 years (dietary)	High selenium intake may not serve as a risk factor for the increase in cataract incidence [98].
Maraini G (2008)	Examine Se effects in the long term	Serum blood test (oral supplement)	Se exhibited different effects on lens [96].
Post M (2018)	To assess Se effect on cataract patients	Serum blood test (dietary)	Low Se levels are associated with cataracts [70].
Zhu X (2014)	Effects of Se on cataract development	Review	Selenium can protect the lens through known as well as unknown mechanisms, which need further investigations [99].
Zhu X (2012)	Se impacts on cataracts	Sodium selenite (oral supplement)	Se may slow the progression of induced cataracts [95].

Some studies evaluated Se levels and the associated effects on retinal conditions in humans (Table 3). For DR, one study showed that a higher oral Se intake was a protective factor [102]. Moreover, another study indicated that selenium had a protecting impact against oxidative stress generated by hyperglycaemia, preserving GPx activity in an RPE cell line [103]. For AMD, a case–control study reported no significant changes in aqueous humour levels of Se between patients with and without AMD, suggesting no correlation [104]. SeNPs could protect against hypoxia-induced oxidative stress on ARPE-19 cells (an RPE cell line) and reduce cell death, possibly via blocking a transient receptor potential cation channel (subfamily M, member 2), which reduced mitochondrial ROS, inflammation, and Ca^2+^ influx [105]. Another study evaluated the influence of SeMet on ARPE-19 and mouse RPE cells and compared cellular levels of GSH. Compared to untreated cells, H_2_O_2_ stressed cells with 1 h of pre-treatment with SeMet showed increased GSH levels due to inducing the cystine/glutamate exchanger SLC7A11 [106]. Finally, a recent review stated that the impact of Se on oxidative stress occurring in retinal pathological conditions remains unclear [107].

Two case–control studies determined that there might be a correlation between glaucoma and selenium levels (Table 4). One revealed that the mean plasma Se level of glaucoma patients (209 ng/mL) was higher than the control (194 ng/mL), suggesting that the level of glutathione peroxides was saturated, leaving an excess of Se in the body, which could show pro-oxidant effects. However, the studies did not offer any statistical analysis to this conclusion. Furthermore, the authors reported a correlation between Se levels in the aqueous humour and glaucoma [108,109]. Other studies demonstrated that there is no significant difference in selenium levels in glaucoma patients compared to controls [60,110].

A study into the effect of total measured selenium on human TM homeostasis revealed that excess Se supplementation may lead to ocular hypertension and therefore glaucoma. This study indicated that Se-caused modifications in cell adhesion and intracellular signalling could impair the filtering of the aqueous humour and regulation of the outflow ability, which could cause a build-up of debris in Schlemm’s canal and increase outflow resistance [111]. In contrast, low selenium levels were correlated with pterygium and uveitis compared to the untreated ischemic group. However, these were single studies for each condition with a limited number of patients each (fewer than 40) [112,113].

Selenium effects were studied on ocular ischemic syndrome patients. The selenium-treated ischemic group (supplemented with oral selenium at 0.1 mg doses twice daily for one month) was compared to the untreated ischemic group in terms of MDA, GSH levels, and ophthalmic examinations. The results showed a significant decrease in MDA (0.45 nmol/mL for the ischemic group vs. 0.06 nmol/mL for the Se-treated group) and an increase in GSH (0.15 nmol/mL for the ischemic group vs. 0.52 nmol/mL for the Se-treated group) [114].

**Table 4:** A summary of Se levels and effects in glaucoma, uveitis, and pterygium patients.

Study	Indication/Goal	Form	Results
Bruhn RL (2009)	Assess relation between glaucoma and Se	Aqueous humour and plasma samples (dietary)	Possible relationship between selenium and glaucoma incidence [108].
Ceylan O (2013)	To evaluate elements in pseudoexfoliation glaucoma (PEXG)	Serum blood test (dietary)	Selenium found to be in a higher concentration in PEXG patients [109].
Conley S (2006)	To characterise how Se affects human TM cell homeostasis	Methyl-selenium (applied to cell culture)	Se causes alteration in protein secretion and cellular adhesion [111].
Dawczysk J (2014)	Assess serum levels of Se in patients with uveitis	Blood samples (dietary)	Decreased selenium levels in chronic patients [113].
Junemann A (2018)	To analyse concentration of Se in primary open-angle glaucoma and PEXG patients against control	Aqueous humour samples (dietary)	No significant difference in Se concentration between patients, but age and gender trends were found [110].
Namuslu M (2013)	To study Se in pterygium tissue	Conjunctiva samples (dietary)	Significant selenium level reduction and study group compared to controls [112].
Telek HH (2019)	To determine the effects of selenium administered for one month on the anterior chamber MDA and GSH levels in patients with ocular ischemic syndrome	Sample from patient ocular anterior chamber (oral supplement)	MDA levels were significantly increased in the ischemia group, while the selenium-supplemented group showed a reduction in MDA levels and a significant increase in GSH values [114].

Most research describes the effects of orally administered Se on ocular conditions. A very limited number of studies described the effects of Se applied topically to the eye [88,92,93]. Oral Se supplementation (organic and inorganic) in the studies mentioned earlier showed antioxidant effects in some cases, while others showed no specific effects, and some showed that oral Se can be a causative factor for some ocular conditions, like selenite-induced cataracts. Organic Se is less toxic than the inorganic form, but it might integrate into other proteins instead of selenoproteins [84].

The toxicity of inorganic selenium has limited its use, and current research focuses on the antioxidant effects of elemental Se [64]. Studies which showed favourable effects describe the administration of Se topically to manage dry eye, so emphasis should be placed on topical administration as a better route than oral administration. Theoretically, topical elemental Se administration can provide a pool to support selenoprotein biosynthesis and GPx expression after being reduced to hydrogen selenide, the active metabolite.

The strategy of prolonging the duration of drug availability on the surface of the eye in order to augment its effectiveness when applied topically has been widely used in various drug delivery methods, leading to the development of a wide range of products [115,116]. The investigation of nanomaterials for use in the eye involves amphiphilic compounds, including liposomes and micelles, as well as polymers like dendrimers and both organic and inorganic nanoparticles [115]. Their high surface-to-volume ratio makes them well suited for delivering therapeutic and targeted molecules [115]. Therefore, using nanoparticles (less than 200 nm) as a platform can potentially help Se to pass through the ocular surface barriers and persist for longer, as well as increase the corneal residence time [115,117].

Lipid-based nanocarriers like liposomes, solid lipid nanoparticles, and micelles are used to solve lipophilic drug solubility issues as well as provide mucoadhesive characteristics without surface decoration. Selenium can be incorporated into a lipid-based nanocarrier, which could be a promising system of delivering selenium topically [118].

Increasing vehicle viscosity is a common way to extend ocular medication residence duration [116]. This is achieved by using synthetic polymers like polyacrylates and polyvinyl alcohols, natural polymers like hyaluronic acid and alginate, and cellulose derivatives. Polymeric excipients show high mucoadhesive properties due to charges (ionic interactions) and functional groups like hydroxyls, amino, and sulfate groups (forming hydrogen bonds) [116]. Furthermore, in situ gelling polymers (temperature-sensitive, pH-sensitive, and ionic-strength-sensitive) can be used to facilitate viscosity and increase residence duration. Different nanoparticle systems can be incorporated into these polymers to increase corneal contact time [115]. Therefore, delivering selenium in a nanoparticle platform using different techniques and polymers to enhance its absorption is a promising approach.

Solid formulations like ocular inserts and contact lenses are other methods of administration that improve ocular bioavailability and drug activity [115,116]. Pharmaceutical technologists have developed nanoparticle-loaded ocular films and contact lenses to extend medication residence duration in the precorneal region while reducing systemic exposure [116].

Hence, more effort is needed in formulating and stabilizing Se for ocular conditions and assessing the outcomes. There is still much to be learned about the relation between Se levels and oxidative stress ocular conditions. Moreover, research into the toxicological properties of selenium nanoparticles is still active, and more studies are needed to fully understand the safety profile.

## 8. Limitations

This study has a number of limitations, the most notable of which is the lack of research articles that directly investigate the antioxidant effects of selenium in the eye. Moreover, there is a relatively small number of clinical investigations studying the direct influence of selenium on eye health. This hinders the capacity to make conclusive determinations. These issues confirm the need for more studies to be performed to allow us to have a deeper understanding of selenium’s function in preserving ocular antioxidant status and its possible consequences for eye health.

## 9. Conclusions

The prevalence of visual impairments associated with ageing in the older population may be largely assigned to several disorders, including cataracts, AMD, glaucoma, and DR. The emergence of these diseases is closely associated with oxidative stress. As humans progress in age, the levels of crucial antioxidants such as glutathione, ascorbic acid, and GPx decline, making the eyes more susceptible to oxidative harm. The effect of oxidative stress is manifested by the induction of LPO and uncontrolled damage to many components of the eye, such as the lens cell membrane, tear lipid layer, retina, and macula. Numerous studies consistently indicate an increase in the levels of ROS biomarkers and a decrease in the activity of antioxidant enzymes, particularly glutathione peroxidase, which is an essential intracellular enzyme that plays a critical role in protecting lipids from peroxidation.

Selenium, a trace element of significant importance, has potential to enhance the production and functionality of selenoproteins and enzymes, particularly GPx. Different types of selenium, including both organic and inorganic forms, have been the subjects of research. Recently, there has been a change in focus towards nanoformulations of selenium owing to their increased effectiveness in boosting GPx function, while also demonstrating reduced toxicity. It is important to note that the excessive consumption of selenium above the prescribed amounts might have a paradoxical effect, as it can stimulate the generation of ROS by creating peroxides. Extensive research has been carried out to investigate the effects of orally administered selenium supplementation on ocular oxidative stress situations. However, the existing body of research demonstrates a notable absence of agreement over the optimum plasma selenium content required for achieving optimal ocular health. 

It is noteworthy that the majority of research on the impact of selenium on ocular health has mostly focused on its oral administration, whereas there has been less investigation into the effects of topically applied selenium. Several studies indicate that topical preparations containing selenium may have promise as candidates for the treatment of ocular diseases associated with oxidative stress. The complex interplay between oxidative stress, the depletion of antioxidants, and the administration of selenium supplements is important in the development of age-related visual impairments. Additional investigation is necessary in order to comprehensively understand the most effective methods of selenium supplementation for the purpose of modifying age-related visual deficits, including both oral and topical administration routes. The use of a diverse range of strategies presents a potential solution for Se in mitigating the detrimental effects of oxidative stress on the ageing ocular system, hence leading to an overall improvement in the wellbeing of the senior demographic.

## Figures and Tables

**Table 1:** Studies on formulations containing Se for ocular surface conditions, showing no ocular irritation and reduced oxidative stress markers.

Study	Indication/Goal	Form	Results
Higuchi A (2010)	To treat dry eye using dry eye rat model and human corneal epithelial cell line	Selenoprotein eye drop (SeP)	Oxidative stress markers were suppressed.GPx activity was increased [88].
Higuchi A (2012)	To treat dry eye using rat model	Se–lactoferrin eye drops	Improved corneal damage caused by dry eye [92].
Higuchi A (2016)	To treat dry eyes	Se–lactoferrin eye drops	No corneal irritation. Increase in corneal uptake by using targeting system [93].
Ou L (2024)	To treat dry eyes	Copper selenide nanoparticles eye drop	The nanoparticles can both activate the NRF2 signalling pathway and enhance the activities of SOD2 and GPX1 to alleviate intracellular ROS [91].

**Table 3:** Studies exploring the effects of dietary selenium on retinal conditions showed various effects, ranging from no effect to a protective effect.

Study	Indication/Goal	Form	Results
Gonzalez R (2018)	Protective effect of Se against induced stress by glucose on retina	Selenite (cell culture study)	Pre-treatment with selenite had the protective effect against induced oxidative stress [103].
Jünemann AGM (2013)	Measure alterations of trace elements levels in aqueous humour of patients with AMD	Aqueous humour samples (dietary)	No significant differences were observed in aqueous humour levels of selenium between patients with and without AMD [104].
She S (2020)	To detect the association of dietary antioxidants and risk of developing DR	Cross-sectional study (dietary)	Higher selenium intake was a protective factor for DR [102].
Ozkaya D (2021)	The protective role of Se by inhibiting hypoxia-induced oxidative stress	SeNPs	SeNPs reversed hypoxia-induced oxidative stress in APRE-19 cells [105].
Zampatti S (2014)	Effects of nutrients on AMD	Review	No recent studies on selenium [107].
Ananth S (2020)	To evaluate the effect of Se on cystine/glutamate exchanger expression and on cellular levels of glutathione	Se-Met (cell culture) and mouse RPE	Se increased GSH levels by inducing cystine/glutamate exchanger expression [106].

## Data Availability

Not applicable.
